# Postoperative urinary retention (POUR) in fast-track total hip and knee arthroplasty: A challenge for orthopedic surgeons

**DOI:** 10.3109/17453674.2014.881683

**Published:** 2014-02-25

**Authors:** Lars S Bjerregaard, Per Bagi, Henrik Kehlet

**Affiliations:** ^1^Section for Surgical Pathophysiology and the Lundbeck Foundation Centre for Fast-track Hip and Knee Replacement;; ^2^Department of Urology, Rigshospitalet Copenhagen University, Copenhagen,Denmark.

Postoperative urine retention (POUR) is a well-known complication in hip (THA) and knee arthroplasty (TKA), but with a variable incidence ranging from 0% to 75% (Balderi and Carli 2010). Although different pharmacological approaches have been attempted to avoid POUR, the results have been inconclusive or controversial (Baldini et al. 2009), leaving indwelling or intermittent bladder catheterization as the only option for prevention and treatment of POUR. However, both POUR and catheterization may increase the risk of urinary tract infection, with subsequent risk of wound and prosthesis infection and/or renal impairment (Madersbacher et al. 2012). Also, POUR and its sequelae may prevent early mobilization, which is essential in fast-track THA and TKA, and may prolong hospitalization and increase re-admission rates (Kehlet 2013). Although fast-track THA and TKA and early mobilization may potentially facilitate restoration of bladder function due to improved pain management with opioid-sparing analgesia, no large-scale data exist on the incidence of POUR and its consequences in fast-track settings. Consequently, POUR should be prioritized by orthopedic surgeons and nurses to develop evidence-based guidelines on prevention and treatment of POUR in fast-track THA and TKA, including efforts to identify patients who are at increased risk of developing POUR.

## The future challenges include the following:

To develop and agree on a precise and unambiguous definition of POUR in order to conduct and compare future clinical trials. Unfortunately, no such definition exists, even among urologists (Kaplan et al. 2008, Baldini et al. 2009). Thus, many studies on POUR do not provide a definition at all and if a definition has been provided, different criteria are used, such as: a bladder volume ranging from 150 to 600 mL, a postoperative need for catheterization (with or without reported indications), inability to void (with or without further details), and/or post-void residual bladder volumes from > 150 mL to > 300 mL (Baldini et al. 2009, Choi et al. 2012). Bladder volume in particular is central for the definition of POUR, since it is the basis for catheterization threshold. The inconsistency is clearly illustrated by Balderi et al. (2011) who, in a retrospective study, defined POUR as the failure to void despite a bladder volume > 500 mL (Balderi et al. 2011), although a review by the same authors 1.5 years previously stated that “…catheterization is recommended at a volume ≥ 600 mL” (Balderi and Carli 2010). In summary, there are no conclusive clinical data on the definition of POUR or on the optimal interventional threshold for catheterization (Madersbacher et al. 2012).

Since POUR may be considered to be an acute, presumed unobstructive urinary retention precipitated by surgery and/or anesthesia (Kaplan et al. 2008), a spontaneous remission may be expected when the precipitating stimuli are gone, which raises the question of risk factors for POUR. Multiple risk factors have been proposed for POUR, and several are relevant in relation to fast-track THA and TKA (Table). The risk factors can be classified as “modifiable” or “non-modifiable”, where the former are individual components of perioperative care that should be optimized according to the fast-track methodology (Kehlet 2013) and the latter are patient-related risk factors to be used in predicting increased risk of developing POUR, and thereby changing postoperative strategies for assessment and treatment. Spinal anesthesia has been advocated to improve outcomes including THA and TKA (Rodgers et al. 2000), but may lead to a transient inhibition of urinary bladder function with the duration and degree of dysfunction depending on the type and dose of local anesthetic and on whether supplementary intrathecal opioids are used (Baldini et al. 2009). Currently, no clear answer is available on the optimal spinal anesthetic technique (Balderi et al. 2011). In contrast to previous recommendations (Fischer et al. 2008), intrathecal opioids should be avoided to reduce the risk of side effects, including POUR (Griesdale et al. 2011). Furthermore, the assumption that spinal anesthesia is superior to general anesthesia in fast-track THA and TKA has recently been questioned (Harsten et al. 2013), calling for reconsideration on the optimal anesthetic technique in this context.

**Table:** Proposed risk factors for postoperative urinary retention after fasttrack total hip and knee arthroplasty

Modifiable
Anesthetic technique
Fluid therapy
Intrathecal opioids
Postoperative analgesic treatment
Indwelling or intermittent bladder catheterization?
Number of intermittent catheterizations before insertion of an indwelling catheter
Unmodifiable
Male sex
High age
Lower urinary tract symptoms
Type of surgery

Postoperative use of systemic opioids increases the risk of POUR (Baldini et al. 2009), and opioid-sparing strategies should be implemented by non-opioid multimodal analgesic approaches and the use of different types of distal local anesthetic blocks.

Historically, it has been recommended that an indwelling catheter should be inserted preoperatively in the operating theater, with removal 1–2 days postoperatively (Iorio et al. 2005), but recent studies have shown that the vast majority of patients undergoing THA and TKA can be handled without an indwelling catheter (Balderi et al. 2011, Karason and Olafsson 2013, Miller et al. 2013). Recently, a randomized controlled trial of intermittent vs. indwelling urinary catheterization in 170 hip surgery patients (hip fracture repair or elective replacement surgery) showed no differences in the incidence of urinary tract infection or in cost-effectiveness (Halleberg et al. 2013), confirming previous inconclusive studies comparing the incidences of urinary tract infection in intermittent and indwelling catheterization (Balderi and Carli 2010). Since prolonged use of indwelling catheters may increase the risk of urinary tract infection (Schaeffer 1986) and hinder early mobilization, it seems rational to use intermittent catheterization to treat POUR in fast-track THA and TKA. A useful tool when using intermittent catheterization is the ultrasound bladder scanner, which has been proven to be valid and which is easy to use by the nurses in the ward (Baldini et al. 2009). However, as discussed above, it needs to be determined who and when to scan. In addition, a widely used, but unproven approach is to place an indwelling catheter if the patient is still incapable of voluntary bladder emptying after 2 intermittent catheterizations (Balderi et al. 2011, Harsten et al. 2013, Karason and Olafsson 2013, Miller et al. 2013), which again calls for future studies on when to use an indwelling catheter. A recent randomized controlled trial by Miller et al. (2013) involving 200 patients found that routine indwelling catheterization was unnecessary in fast-track THA using spinal anesthesia and non-opioid, multimodal analgesia. However, the study excluded patients with confirmed renal disease and/or previous urogenital surgery, who must be considered to be at high risk of developing POUR.

High age and male sex are unmodifiable risk factors for development of POUR, which is probably explained by a higher incidence of lower urinary tract symptoms (LUTS) (Baldini et al. 2009). As a consequence (but not based on evidence), age > 70 years has been suggested as a useful “cutoff” value for increased risk of POUR in men (Sarasin et al. 2006). Since the International Prostate Symptom Score (IPSS) can be used to quantify lower urinary tract symptoms in both men and women, it has been proposed for use in predicting the risk of POUR, but to date the studies have been heterogeneous regarding sample size, study design, and statistical approach—and are thereby inconclusive (Elkhodair et al. 2005, Sarasin et al. 2006, Kieffer and Kane 2012). However, the use of IPSS to predict POUR in fast-track THA and TKA is rational, calling for high-quality studies in standardized settings with well-defined criteria for POUR. Also, we still need to know whether THA carries a higher risk of POUR than TKA (Balderi et al. 2011, Griesdale et al. 2011, Madersbacher et al. 2012).

Finally, our current knowledge about POUR and its short- and long-term consequences in fast-track THA and TKA is limited, especially regarding long-term sequelae such as infections, detrusor damage, and subsequent atonic bladder (Baldini et al. 2009, Madersbacher et al. 2012).

In summary, elimination and/or treatment of POUR in fast-track THA and TKA constitutes a challenge for orthopedic surgeons and nurses. Although editorials and reviews often call for further studies, POUR is such a common problem that the clinically relevant questions regarding definition, risk factors, consequences, and treatment strategies should be relatively easy to answer in these high-volume operations.
